# A New Tribe of the Ladybird Beetle Subfamily Microweiseinae (Coleoptera: Coccinellidae) Discovered on an Island in the North Atlantic Ocean

**DOI:** 10.3390/insects11060367

**Published:** 2020-06-13

**Authors:** Karol Szawaryn, Jaroslav Větrovec, Wioletta Tomaszewska

**Affiliations:** 1 Museum and Institute of Zoology, Polish Academy of Sciences, Wilcza 64, 00-679 Warsaw, Poland; 2 Buzulucká 1105, 500 03 Hradec Králové, Czech Republic; JerryVetrak@seznam.cz

**Keywords:** Coccinelloidea, lady beetles, Macaronesia, Madeira, Microweiseinae, new genus, new tribe, Portugal

## Abstract

Microweiseinae is a quite recently established subfamily within ladybird beetles (Coccinellidae). According to recent analyses of morphological and molecular data, it has been divided into three tribes. Members of the subfamily are distributed mostly in tropical and subtropical regions of the world. Despite several recent taxonomical studies of this group, its diversity and distribution is still not fully understood. Recent field collecting on Madeira Island resulted in the discovery of interesting specimens belonging to a yet unknown taxon, described here as *Madeirodula atlantica* **gen. et sp. nov.** Phylogenetic analysis of morphological characters indicate that the new taxon form a distinct branch within the subfamily Microweiseinae, for which we propose a new tribe Madeirodulini **trib. nov.** Evolutionary trends within the subfamily are discussed, and an updated key to the tribes of Microweiseinae is provided.

## 1. Introduction

Recent investigations of the Coccinellidae phylogeny significantly changed the classification of this group of beetles. Based on morphological data, Ślipiński [1] proposed dividing Coccinellidae into just two subfamilies, Microweiseinae and Coccinellinae, instead of six or seven previously proposed [2,3,4]. This point of view was later confirmed by subsequent molecular studies (e.g., [5,6]).

Members of the subfamily Microweiseinae have cryptic coloration and a very small body size, unlike most of the commonly known ladybird beetles with contrasting white, red, and black aposematic coloration. Their small, brown colored, pubescent body forms, more closely resemble some Anamorphidae, Eupsilobiidae, or Corylophidae than ‘true’ lady beetles. Recent molecular study of the superfamily Cucujoidea [6] places Microweiseinae as an intermediate clade between the remaining Coccinellidae and Endomychidae sensu lato (handsome fungus beetles).

Microweiseinae are regarded as a more primitive group of Coccinellidae, inhabiting mainly leaf litter or under bark habitats. Members of Microweiseinae inhabit mostly tropical and subtropical zones of the world, however, their distribution and life histories are largely unknown, and still many new species are described from different parts of the world [7,8,9,10,11]. The oldest representatives of this group are known from Eocene Baltic amber and belong to the tribe Serangiini [12,13].

Modern classification of Microweiseinae based on phylogenetic analysis of morphological characters [14] recognizes three tribes: Carinodulini Gordon et al., Serangiini Pope, and Microweiseini Leng, containing together about 150 species classified until the present study in 22 genera. The monophyly of the subfamily is well supported by several morphological characters of which the placement of antennal insertions well before the anterior margin of eyes, and asymmetrical tegmen of the male genitalia are unique modifications within the whole family Coccinellidae. Each tribe of Microweiseinae is quite distinct morphologically. The most apparent characters defining the tribes are: for Carinodulini, sublateral carinae on pronotum often associated with pits, and the V-shaped metaventral postcoxal lines; for Serangiini, mandible with a long dorsal process, antennal club large one-segmented and flattened, the prosternum raised and forming a triangular plate, and the ventral side of the body with distinct impressions for reception of legs; and for Microweiseini (the most diverse group of the subfamily), glandular structures on the subgenal area well delimited and separated from the mouth cavity and abdominal postcoxal lines descending and incomplete (although numerous exceptions/reversals of characters states are present in this tribe) [14].

The present study was inspired by a discovery of unusual new genus of the subfamily Microweiseinae, which was found on Madeira Island. It is described here as *Madeirodula* gen. nov., along with *M. atlantica* sp. nov. This is the first member of the Microweiseinae native to Europe (in terms of administrative boundaries), and it possesses a mixture of morphological characters present in all known tribes, especially of Carinodulini and Microweiseini. To test the systematic position of the new genus within the subfamily, a phylogenetic analysis was performed.

## 2. Materials and Methods

### 2.1. Type Specimen Deposition and Measurements

Specimens examined during this study were deposited in the following collections: NMPC, Natural History Museum Prague, Czechia, and JVC, the private collection of Jaroslav Větrovec, Hradec Králové, Czechia.

Measurements of the body structures were recorded as follows [15]: TL—Total body length, from the apical margin of clypeus to the apex of elytra; PL—Pronotal length, from the middle of the anterior margin to the margin of basal foramen; PW—Pronotal width in the widest part; EL—Elytral length along the suture, including scutellar shield; EW—Elytral width across both elytra in the widest part. Male genitalia were dissected, cleared in 10% KOH solution, and subsequently transferred in glycerol on slide for further study. After examination, the genitalia were glued on cards and pinned beneath the specimen. Digital photographs were made using a Leica MZ 16 stereo microscope with a digital camera IC 3D attached. Terminology used in this paper follows Ślipiński [1] and Lawrence et al. [16].

### 2.2. Taxon Sampling and Morphology Coding

Taxon sampling and characters used for the phylogenetic analysis based on Escalona and Ślipiński [14], with terminals being the results of that study (considering the resulted synonyms). Additionally, two recently described genera of Microweiseinae were added to the dataset: *Pangia* Wang & Ren [8] and *Ruthmuelleria* Jałoszyński & Ślipiński [10]. The list of characters from Escalona and Ślipiński [14] was expanded with three additional characters. As a result, in our data matrix (Supplementary File S1), 22 currently recognized genera of the subfamily Microweiseinae and a new genus described here were scored for 48 multistate characters using DELTA (DEscription Language for TAxonomy) [17]. Two genera of Coccinellinae (*Rhyzobius* Stephens, *Sticholotis* Crotch) and one member of the family Corylophidae (*Holopsis* Broun) were coded as outgroups.

### 2.3. Characters Used in the Analyses

1. Anterior edge of clypeus: (1) smooth; (2) margined.

2. Frontoclypeus in front of eyes: (1) straight or weakly emarginate; (2) distinctly emarginate.

3. Distance between antennal insertions: (1) less than that between inner eye margins; (2) equal or greater than that between inner eye margins.

4. Posterior margin of antennal socket: (1) distinctly in front of eyes; (2) adjacent to anterior eye margin or in between eyes.

5. Supraorbital sulcus: (1) absent; (2) present.

6. Supraorbital marginal line: (1) absent; (2) present.

7. Occipital endocarina: (1) present; (2) absent.

8. Ligula: (1) sclerotized; (2) membranous anteriorly.

9. Distance between insertions of labial palps: (1) at least as great as the width of the palpifer; (2) less than the width of the palpifer or palps contiguous.

10. Apical margin of mentum: (1) truncate; (2) shallowly emarginate; (3) deeply emarginate.

11. Maxillary galea: (1) absent; (2) present.

12. Maxillary stipes: (1) divided into basistipes and mediostipes; (2) fused into single, elongate structure.

13. Maxillary palpifer: (1) convex, not receiving maxillary palp in repose; (2) foveate externally to receive maxillary palp in repose.

14. Shape of terminal maxillary palpomere: (1) knife-like (cultriform); (2) oval and broadened medially; (3) conical or parallel sided.

15. External border of mandible dorsally: (1) normal, broadly rounded; (2) projected into a process.

16. Mandibular apex: (1) bidentate; (2) unidentate; (3) broadly rounded, reduced.

17. Mandibular mola: (1) developed but smooth, without grinding surfaces or molar tooth; (2) with grinding surfaces or molar tooth.

18. Gena ventrally: (1) extending forwards and forming a frame for mouthparts, approaching clypeus from below; (2) extending forwards and forming a frame for mouthparts, and entirely fused to clypeus in front of antennal insertions; (3) not extending forwards and clearly separated from clypeus.

19. Glandular structures or glandular openings on subgenal area: (1) well delimited and separated from the mouth cavity; (2) not well delimited and closely adjacent to the mouth cavity; (3) absent.

20. Number of antennal segments: (1) 7–10; (2) 11.

21. Antennal club: (1) one-segmented and distinctly flattened; (2) one-segmented and rounded in cross section; (3) two- to four-segmented.

22. Anterior edge of pronotum: (1) medially emarginate; (2) arcuate.

23. Sublateral carina on pronotum: (1) absent; (2) at least partially visible.

24. Anterior corners of pronotum: (1) separated from disc by line joined to lateral and anterior margins; (2) separated from disc by a line joined to anterior margin only; (3) not separated from disc.

25. Pits on pronotum: (1) absent; (2) present.

26. Pits on prosternum: (1) absent; (2) present.

27. Prosternum in front of coxa: (1) strongly reduced; (2) well developed and always longer than half of coxa length.

28. Prosternum: (1) entirely raised, forming a triangular plate; (2) with anteromedian prominence (chinpiece as defined by Lawrence et al., 2011); (3) straight anteriorly, without chinpiece; (4) anterior margin broadly arcuate, emarginate laterally to receive antennal funicle in repose.

29. Prosternal process: (1) broad and extending behind coxa; (2) narrow, carina-like and often incomplete posteriorly.

30. Procoxal cavities: (1) with lateral slits; (2) without slits.

31. Prosternal rest: (1) absent; (2) present.

32. Anterior margin of mesoventrite: (1) about same level as mesometaventral junction; (2) on much lower level than the mesometaventral junction.

33. Mesometaventral junction: (1) narrow, as wide or less than a coxal diameter; (2) broad, distinctly broader than a coxal diameter.

34. Metaventral postcoxal lines: (1) joined medially; (2) separate medially.

35. Metaventral postcoxal lines: (1) V-shaped; (2) descending laterally; (3) recurved.

36. Metaventral postcoxal lines and associated crural impressions: (1) reaching metanepisternum; (2) limited to metaventrite only.

37. Number of abdominal ventrites: (1) 5; (2) 6.

38. Abdominal postcoxal lines: (1) V-shaped; (2) descending and incomplete, not reaching lateral edge; (3) descending and complete, reaching lateral edge; (4) recurved.

39. Accessory postcoxal line: (1) absent; (2) present.

40. Elytral epipleuron: (1) without foveae receiving legs in repose; (2) with foveae receiving legs in repose.

41. Number of tarsomeres: (1) three; (2) four.

42. Pretarsal claws: (1) simple; (2) appendiculate.

43. Male genitalia with tegmen (at least phallobase): (1) asymmetrical; (2) symmetrical.

44. Number of abdominal spiracles: (1) 7; (2) 5.

45. Penis base: (1) without capsule; (2) with distinct capsule.

46. Pronotum with posterior border: (1) absent; (2) present.

47. Round imprints on elytral surface: (1) absent, (2) present (Figure 4C).

48. Elytral setation: (1) single, (2) double (Figure 4C), (3) absent.

### 2.4. Phylogenetic Analyses

The matrix was exported to the Nexus format, checked in Mesquite [18] and exported to the TNT format for phylogenetic analyses. Unknown character states were coded with ‘?’. The maximum parsimony (MP) analyses were conducted in TNT 1.5 [19] using the Traditional Search option to find the most parsimonious trees (MPTs) under the following parameters: memory set to hold 1,000,000 trees, tree bisection—reconnection (TBR) branch-swapping algorithm with 1000 replications saving 10 trees per replicate; zero-length branches collapsed after the search, with implied weighting option with a k value set to 3. Bremer support was calculated using the TNT Bremer function, using suboptimal trees up to 20 steps longer. Character mapping was done in Winclada v1.00.08 [20] using unambiguous optimization. All characters were treated as unordered and analyses were performed under equal weights. The analysis was set to find the minimum tree length.

Additionally, the Bayesian inference (BI) was conducted in MrBayes v3.2.6 [21] running on the CIPRES Science Gateway v3.3. (phylo.org), using the Mkv model for standard data. All analyses used four chains (one cold and three heated) and two runs of 10 million generations. Autapomorphies were included in the dataset, and the analyses were conducted using a gamma distribution. Convergence of the two runs was visualized in Tracer v1.6 [22], and by examining potential scale reduction factor (PSRF) values and the average standard deviation of split frequencies in the MrBayes output.

## 3. Results

### 3.1. Phylogenetic Analyses

**Figure 1:**
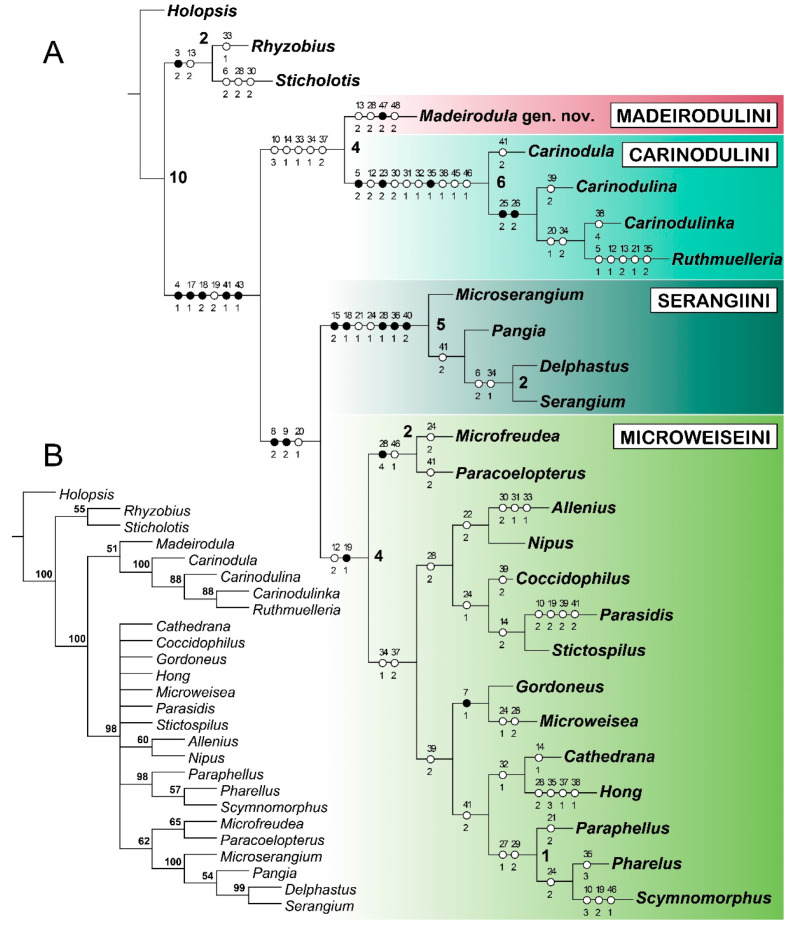
Results of the phylogenetic analyses. (**A**) The single most parsimonious tree from TNT, with character evolution of Microweiseinae; all character states are treated as unordered and equally weighted. Characters were mapped on branches using unambiguous character changes in Winclada (black circles, non-homoplasious changes; white circles, homoplasious changes); numbers above the circles indicate characters, and numbers below circles indicate their states. Numbers at the corresponding nodes showing Bremer support values. (**B**) Consensus tree from the Bayesian analysis, with numbers at the corresponding nodes showing posterior probabilities reported for the corresponding nodes.

The MP analysis under Traditional Search (MP TS) resulted in a single most parsimonious tree with topology parameters (L = 142; CI = 42; RI = 70) (Figure 1A). The topology of the tree is similar to that presented as the preferred tree in Escalona and Ślipiński [14], with all known tribes of Microweiseinae: Carinodulini, Serangiini, Microweiseini, recovered. The differences refer to internal relationships between genera within the tribe Microweiseini, however, with *Microfreudea* Fürsch + *Paracoelopterus* Normand recovered as sister groups, and in the same position on the tree (as sister to the rest of Microweiseini) in both studies. In our BI analysis (Figure 1B) Carinodulini are also recovered as a distinct clade, but Serangiini are embedded in Microweiseini and both tribes form a single clade with unresolved internal relationships. Interestingly, this placement of Serangiini in our BI analysis agrees with some variants of parsimony analysis from Escalona and Ślipiński [14]. In both present analyses (MP, BI), the new genus described here as *Madeirodula* gen. nov. was recovered as a sister taxon to the tribe Carinodulini, with its own apomorphies, enabling us to propose a new tribe of Microweiseinae.

### 3.2. Taxonomy

Order: Coleoptera Linnaeus, 1758.

Family: Coccinellidae Latreille, 1807.

Subfamily: Microweiseinae Leng, 1920.

**Figure 2:**
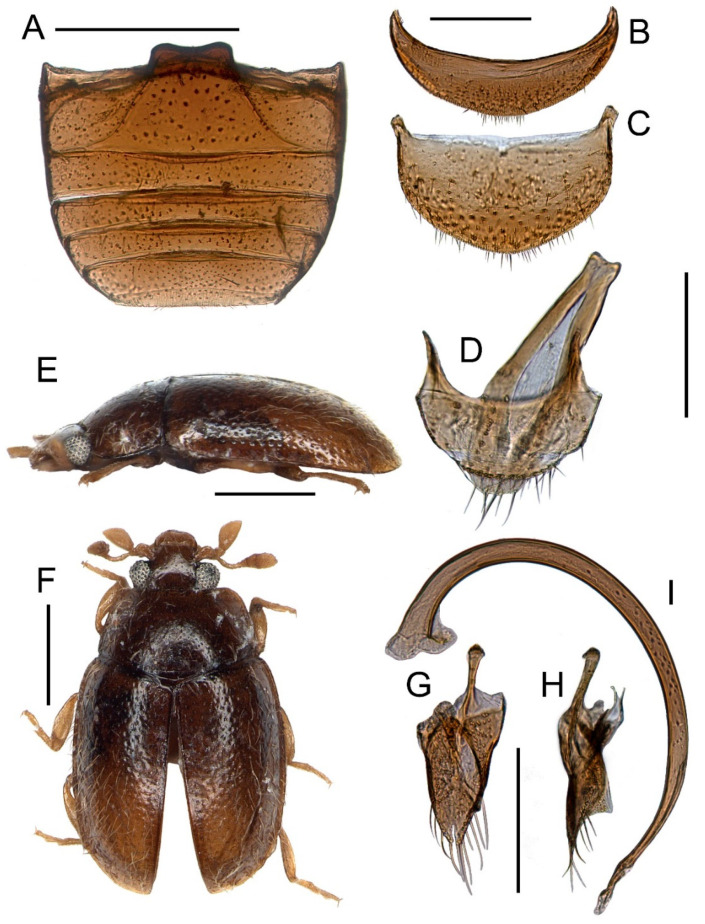
Morphology of *Madeirodula atlantica* gen. et sp. nov. (**A**) abdomen. (**B**) ventrite 6 (sternite VIII). (**C**) tergite VIII. (**D**) male abdominal segments IX and X. (**E**) habitus, lateral. (**F**) habitus, dorsal. (**G**) tegmen, inner. (**H**) tegmen, lateral. (**I**) penis, lateral. Scale bars: 0.5 mm (**A**,**E**,**F**); 0.2 mm (**B**–**D**,**G**–**I**).

**Figure 3:**
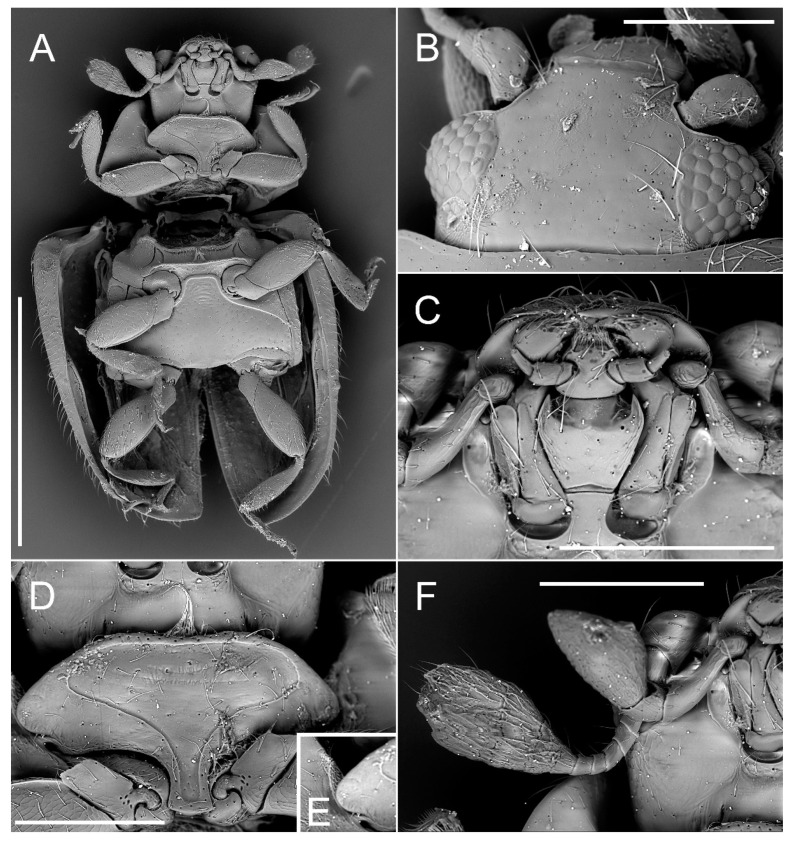
Morphology of *Madeirodula atlantica* gen. et sp. nov. (**A**) habitus, ventral. (**B**) head, dorsal. (**C**) mouthparts, ventral. (**D**) gular region of head and prosternum, (**E**) antero-median part of hypomeron with area of glandular structures. (**F**) maxillary palp and antenna. Scale bars: 1 mm (**A**); 0.2 mm (**B**–**F**).

**Figure 4:**
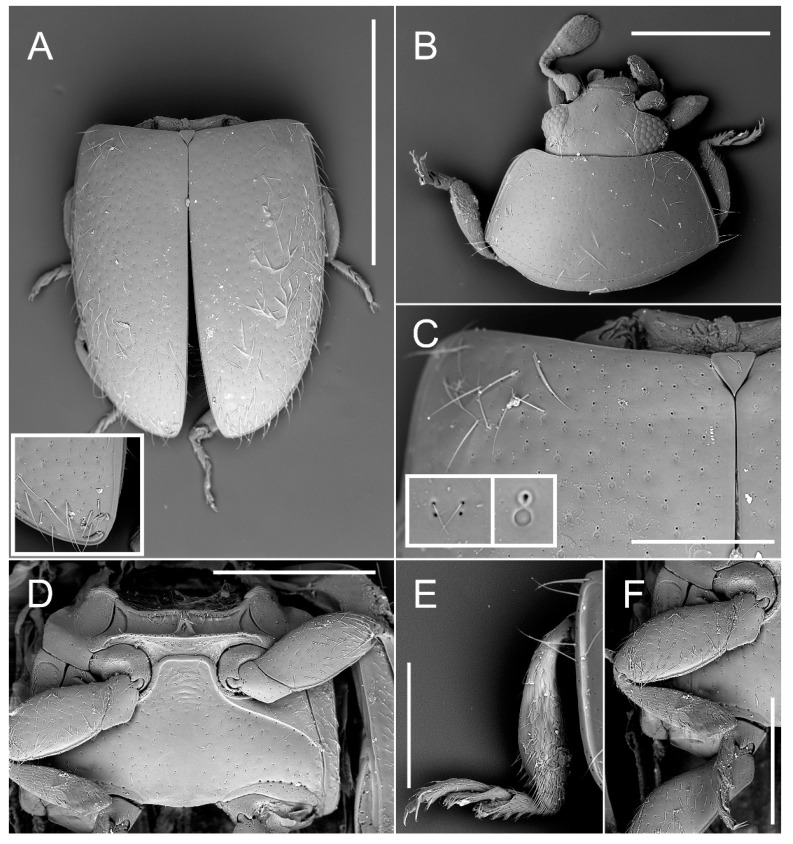
Morphology of *Madeirodula atlantica* gen. et sp. nov. (**A**) elytra, dorsal with enlarged apex with sutural line. (**B**) head an pronotum, dorsal. (**C**) base of elytra and scutellar shield with enlarged details of elytral surface structures. (**D**) meso- and metaventrite. (**E**) left mid-leg, dorsal. (**F**) right mid-leg, ventral. Scale bars: 1 mm (**A**); 0.5 mm (**B**); 0.4 mm (**D**); 0.2 mm (**C**,**E**,**F**).

Tribe: Madeirodulini trib. nov. (Figure 2, Figure 3 and Figure 4).

Type genus. *Madeirodula* gen. nov., by monotypy and present designation.

ZooBank: urn:lsid:zoobank.org:act:B0157299-987D-4A22-8C62-E6D7B9AF6498

Etymology. The tribal name is derived from the name of a type genus.

Diagnosis. The new tribe Madeirodulini resembles members of the tribe Microweiseini in general body shape, but it can be separated from them by having a bidentate mandibular apex (vs. unidentate), antennae consisting of 11 antennomeres (vs. 7–10), cultriform apical maxillary palpomere (vs. conical, parallel sided or rounded), paired apophyses of male sternum IX joined apically in form of inverted V (vs. inverted Y), and by a lack of line separating anterior corners of pronotum from disc. Bidentate mandibles, antennae with 11 antennomeres, and cultriform apical maxillary palpomere are shared with members of the tribe Carinodulini, however, Madeirodulini can be separated from Carinodulini by the prosternum forming a large chinpiece, abdominal postcoxal lines descending and incomplete laterally (vs. U- or V-shaped in Carinodulini), well developed hind wings, and by a lack of lateral carinae or pits on pronotum. From Serangiini, Madeirodulini can be distinguished by having an antennal club consisting of three antennomeres (vs. one antennomere), the mandible with broadly rounded, simple external border (vs. projected into a process in Serangiini), and the elytral epipleura without foveae for reception of legs.

Genus: *Madeirodula* gen. nov. (Figure 2, Figure 3 and Figure 4).

Type species. *Madeirodula atlantica* sp. nov., by monotypy and present designation.

ZooBank: urn:lsid:zoobank.org:act: A09A18D3-BA7C-41BD-8F77-0D08A4027186

Etymology. First part of the genus name is derived from the name of the island where the type specimens were collected, and the second part refers to *Carinodula*, the type genus of Carinodulini, sister group of the new tribe.

Diagnosis. Same as for the tribe.

Description. Body elongate oval, flattened; dorsal surface pubescent (Figure 2E,F).

Head transverse, with eyes large, coarsely faceted (Figure 3B). Frontoclypeus with lateral edges margined, emarginate around antennal insertions with distinct ridge; without supraorbital sulcus. Subantennal grooves distinct, long, reaching base of maxillary stipes (Figure 3C). Subgenal gland openings present. Gula elongate, surface smooth with few apical setae, gular sutures distinct (Figure 3D). Antenna consists of 11 antennomeres with three last antennomeres forming a distinct, large club (Figure 3F and Figure 4B). Mandibles with apex bidentate (Figure 3C). Maxillary stipes divided into basistipes and mediostipes (Figure 3C), cardo semicircular; maxillary palpifer foveate externally to receive maxillary palp in repose; galea large, well developed, rounded; lacinia small, elongate, with several stiff setae at apex; second maxillary palpomere elongate, about 2.5 times longer than the third; terminal maxillary palpomere large, knife-like (cultriform) (Figure 3F). Submentum (Figure 3C) moderately broad, distinctly narrower than maxillary cavity, slightly broadening toward apical part; mentum trapezoidal, broadening toward apex, with apical margin deeply emarginate; whole prementum well sclerotized, shorter than labial palp, and apex fringed with setae; labial palps separated with a distance of about the width of basal palpomere; second palpomere large, broad, about twice as broad as the terminal palpomere; terminal palpomere convergent apically.

Prothorax. Pronotum transverse, without pits, with lateral sides narrowly and base comparatively widely margined; anterior margin with lines/bordering only in anterior angles, and median part not margined (Figure 4B). Pronotal disc convex, covered with punctures of double size, larger punctures bearing large seta, sometimes with additional small puncture at its base; smaller punctures bearing small seta with second small puncture at its base; lateral edge smooth, sublateral carina absent (Figure 4B). Prosternum with large, expanded laterally chinpiece (Figure 3D); prosternal process parallel-sided, with rounded apex; prosternal carinae present, continuing along prosternal chinpiece and forming anterior prosternal border. Antero-median part of hypomeron with area of glandular structures or sensilla (Figure 3E). Procoxal cavities transverse, with lateral slits (Figure 3A).

Pterothorax. Elytra irregularly punctate with punctures of double size (Figure 4A,C); larger punctures bearing large seta with additional large round impression at their base; smaller punctures bearing small seta with second small puncture at base; sutural line present only in apical part (Figure 4A). Epipleura incomplete, narrow, without foveae, with short border line just in the mid length (Figure 3A). Metathoracic wings well developed. Scutellar shield triangular, about as long as its width (Figure 4C). Mesoventrite transverse (Figure 4D), flat; with row of pores present on anterior raised margin; procoxal rest present; meso-metaventral junction arcuate anteriorly; at midline slightly broader than mid coxa. Metaventrite transverse (Figure 4D), longer than ventrite 1; metaventral postcoxal lines joined medially at metaventral process forming straight line, laterally complete, descending; Metaventrite with rows of pores present under postcoxal lines and above hind coxae. Surface of metaventrite covered with small, sparsely distributed paired punctures (rarely single).

Abdomen with six ventrites (Figure 2A); ventrite 1 about as long as ventrites 2–4 combined; postcoxal lines incomplete, not recurved, reaching posterior margin of the abdominal ventrite 1; ventrite 5 truncate apically, ventrite 6 rounded (Figure 2B), tergite VIII arcuate (Figure 2C).

Legs slender (Figure 3A and Figure 4F); coxae sub-rectangular with rounded inner angle, with a group of small pore openings at basal part; femora slightly swollen; tibiae without apical spurs; tarsi with three tarsomeres (Figure 4E); tarsal claws with large basal, rectangular tooth.

Male terminalia and genitalia. Segment 9 with tergite and sternite not fused laterally (not forming a capsule), connected by transparent membrane (Figure 2D); sternite IX narrow, elongate, rounded apically, with paired apophyses, broad and rod-like, joined apically; tergite IX transverse, truncate at apex. Tergite X small, transverse, rounded apically, connected to tergite IX by a transparent membrane (Figure 2D). Tegmen asymmetrical (Figure 2G,H); parameres fused medially with short notch at apex; penis guide asymmetrical, reduced; tegminal strut short, simple, slightly widened at apex. Penis elongate and curved (Figure 2I), with basal capsule weakly developed but distinct.

**Figure 5:**
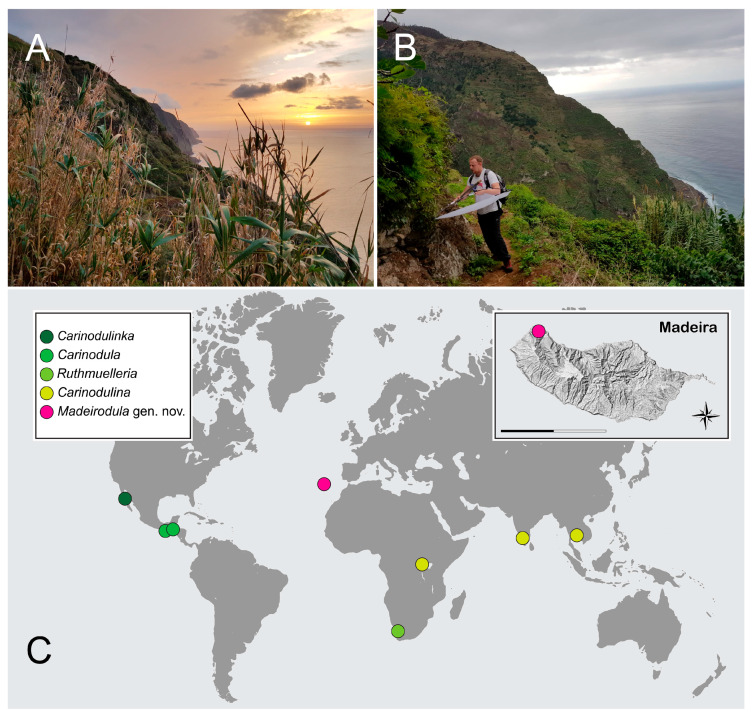
(**A**,**B**) Type locality and habitat where type specimens were captured. (**C**) geographic distribution of Carinodulini and Madeirodulini taxa, with indication of the type locality on Madeira Island. Scale bar: 30 km.

Distribution. Europe, Macaronesia, Madeira (Figure 5C).

*Madeirodula atlantica* sp. nov. (Figure 2, Figure 3 and Figure 4).

ZooBank: urn:lsid:zoobank.org:act: 483232A5-650A-4567-B4EE-FD6B68DF6EB1

Etymology. The specific name refers to the Atlantic Ocean.

Type material. Holotype, male (NMPC); Madeira, 16.11.2017, Santa Maria Madalena, Pombais, costal slopes, 32°51′31.8″N 17°12′10.3″W, 400 m, lgt. J. Větrovec. Paratype, male (JVC); same data as holotype.

Diagnosis. Same as for the genus.

Description. Length = 1.80 mm; width = 1.05 mm; TL/EW = 1.71; PL/PW = 0.59; PL/EL = 0.39; PW/EW = 0.79; EL/EW = 1.20. Body elongate, flattened (Figure 2E,F), covered with double size setae, well visible long sparse setae, and very short and delicate setae that can be observed only under high magnification (Figure 4C). Color chestnut brown, legs and mouthparts more pale. Eyes large, prominent, extending well beyond head capsule.

Antenna with 11 antennomeres; scape large, swollen; pedicel barrel shaped; antennomere 3 elongate; antennomeres 4–5 subquadrate; antennomeres 6–7 transverse; antennomere 8 trapezoidal, apically broadened; antennomeres 9–11 forming distinct, rounded club, antennomeres 9 and 10 transverse, 11 large, about as long as wide.

Abdomen with six ventrites in male (Figure 2A); ventrite 1 about as long as 2–4 combined, ventrites 2–4 equal in length, ventrite 5 about 1.5 times longer than ventrite 4; ventrite 6 narrow, rounded apically (Figure 2B); tergite VIII large, arcuate (Figure 2C).

Male genitalia. Tegmen asymmetrical with oblique cavity for reception of penis (Figure 2G,H), parameres fused medially with rows of long setae at outer margin, penis guide with apical notch. Penis well sclerotized (Figure 2I), elongate and curved, with membranous apex, and with basal capsule weakly developed.

Female unknown.

Habitat. The two specimens were swept from the vegetation that grows on the northern slopes of Madeira Island (Figure 5A,B), during the evening. Specimens were beaten from *Arundo donax* L. grass. The biology is unknown.

### 3.3. Updated Key to the Tribes of Microweiseinae

Modified from Escalona and Ślipiński [14].

1. Mandibular apex bidentate; terminal maxillary palpomere knife-like (cultriform) …………. 2.

– Mandibular apex unidentate; terminal maxillary palpomere variable but never distinctly knife-like ………………………………………..………..………………………………………………………… 3.

2. Prosternum with straight anterior border, without chinpiece; pronotum with sublateral carina well separated from lateral margin; prothorax and mesoventrite usually with deep pits (except *Carinodula*); abdominal postcoxal lines recurved, complete, V- or U-shaped; hind wings absent ……………………………………………….………………………………………………… Carinodulini.

– Prosternum with distinct chinpiece; pronotum only narrowly bordered laterally, without carina; prothorax and mesoventrite without pits; abdominal postcoxal lines reaching posterior margin of the abdominal ventrite 1, not recurved, incomplete; hind wings present ……… ………………………………………………………………………………………Madeirodulini trib. nov.

3. Prosternum elevated, forming large triangular plate, its anterior margin closes with anterior margin of clypeus in repose; ventral side of the body, including epipleura deeply foveate, receiving folded legs; abdomen with 5 ventrites, ventrite 5 as long as ventrites 2–4 combined; antenna composed of eight or nine antennomeres with flattened one-antennomere club ………….… ………………………………………………………………………………………………………Serangiini.

– Prosternum variable, flat, strongly reduced, or variously lobed anteriorly, partially or almost completely concealing mouthparts; ventral side of the body including epipleura without foveae receiving folded legs; abdomen with five or six ventrites, ventrite 5 almost always short; antenna composed of eight to ten antennomeres with one to three-antennomeres club that is circular in cross section …………………………………………………………………………………………Microweiseini.

## 4. Discussion

Despite the close external similarity of *Madeirodula* gen. nov. to members of Microweiseini, our phylogenetic analysis recovered the new genus as a sister group to the tribe Carinodulini (Figure 1A). This sister relationship is supported by the set of homolpastic characters: apical margin of mentum deeply emarginate (10:3); terminal maxillary palpomere knife-like (cultriform) (14:1); mesometaventral junction narrow, as wide or less than a coxal diameter (33:1); metaventral postcoxal lines joined medially (34:1); and six abdominal ventrites (37:1). *Madeirodula* also shares with Carinodulini a bidentate mandibular apex. However, no synapomorphy characteristic for Crinodulini is present in *Madeirodula* (i.e., supraorbital sulcus present (5:2) (vs. absent in *Madeirodula*); sublateral carina on pronotum at least partially visible (23:2) (vs. absent); and metaventral postcoxal lines V-shaped (35:1) (vs. descending)). Moreover, grouping of *Madeirodula* gen. nov. with Caridnodulini was recovered in our BI analysis with low support (PP = 51), so this sister relationship should be treated as preliminary and inconclusive.

Our analysis recovered *Madeirodula* gen. nov. as a separate, monophyletic lineage, proposed here as a new tribe, supported by one apomorphy: elytra with characteristic pattern/double elytral punctuation (47:2), and by the following homoplastic characters: maxillary palpifer foveate externally to receive maxillary palp in repose (13:2); prosternum with chinpiece (28:2); and elytra with double setation (character 48:2).

*Madeirodula* gen. nov. also possesses a very unique structure of the male terminalia and genitalia. Sclerites of the abdominal segment IX (tergite and sternite) are not fused (Figure 2D), as in most ladybird beetles, and are of distinctly different size: tergite is transverse and large, while sternite is comparatively narrow and elongate (in other coccinellids both plates are about the same size and shape). Moreover, sternite IX of *Madeirodula* gen. nov. bears paired apophyses, two rod-like sclerites joined apically in the form of an inverted V-shape (Figure 2D). In other Microweiseinae, there are also paired apophyses, but clearly form an inverted Y-shape (with a single branch in apical part and forked at the base [14]), while in most members of the subfamily Coccinellinae, a single rod-like apophysis is present, or sometimes it is completely reduced [23,24]. The structure of apophyses of the male genital segment in *Madeirodula* gen. nov. is similar to the condition present in some handsome fungus beetles (Endomychidae sensu lato) [25], and can be regarded as a more primitive form. The asymmetrical tegmen in *Madeirodula* gen. nov. is of a typical form for the members of Microweiseinae, however, strongly reduced penis guide as well as fused parameres with only a small apical notch separating them is unusual and support distinct evolutionary trends in this lineage. In most remaining Microweisenae, penis guide is elongate with parameres separated and of equal length [8,14], or even completely reduced with only penis guide present, like in Carinodulini [26,27].

Possessing a mixture of morphological characters present in both Carinodulini and Microweiseini, *Madeirodula* gen. nov. can be considered an intermediate taxon between both tribes. On the other hand, the unique structure of the male terminalia shows more primordial condition than in other tribes of Microweseinae. Further analyses including molecular data have to be conducted to resolve the placement of *Madeirodula* gen. nov. in the classification of Microweiseinae.

Nothing is known about the biology and ecology of *Madeirodula atlantica* sp. nov., but large, prominent eyes (Figure 2F and Figure 3B) and the fact that specimens were collected during the evening when the sun was going down (Figure 5A,B), may suggest its nocturnal lifestyle.

Members of Microweiseinae are distributed worldwide, with most species occurring in tropical and subtropical regions. Both Serangiini and Microweiseini are distributed pantropically. Carinodulini shows a very interesting distribution pattern with species known from isolated, relictual, mountainous areas in Africa, Asia, and Central and North America. Madeirodulini, discovered on Madeira Island, seemed to ‘match’ this interesting scattered pattern of distribution (Figure 5C). However, without thorough investigation of the historical biogeography of the Microweiseinae, it is hard to understand what processes shaped the present distribution pattern of the subfamily, and especially of Carinodulini + Madeirodulini lineage.

## 5. Conclusions

The recent field collecting on the Portuguese island of Madeira in the North Atlantic Ocean resulted in the discovery of a new genus and species belonging to the ladybird beetle subfamily Microweiseinae.

Microweiseinae, unlike most coccinellids, have cryptic coloration and a very small body size, rather resembling members of other Coccinelloidea families (Anamorphidae, Eupsilobiidae, or Corylophidae) than ‘true’ lady beetles. They inhabit mainly leaf litter or under bark habitats and are regarded as a more primitive group of Coccinellidae. Despite several recent taxonomical studies of this group, its diversity and distribution is still largely unknown. The first member of Microweiseinae found in the southwestern ends of the Palaearctic region is described here as *Madeirodula atlantica* gen. et sp. nov. Its morphology, being a mixture of morphological characters from all known tribes, was the basis for conducting the phylogenetic analysis to test the systematic position of the new genus within the subfamily.

Phylogenetic analysis recovered *Madeirodula* as a distinct evolutionary lineage within the subfamily Microweiseinae, proposed here as Madeirodulini trib. nov., sister to Carinodulini. Besides the shared morphological characters, both tribes displayed an unusual, very interesting “scattered” pattern of distribution, inhabiting isolated mountainous areas in tropical and subtropical regions around the world. This pattern of the present distribution does not allow, however, to make any conclusions regarding a possible area of origin of the ancestor of Carinodulini + Madeirodulini lineage and subsequent ways of its diversification. Further taxonomical efforts and comprehensive study of the historical biogeography of the entire subfamily Microweiseinae is needed to shed more light into the evolution of this group of lady beetles that have a cryptic appearance and mode of life.

## Supplementary Materials

The following are available online at https://www.mdpi.com/2075-4450/11/6/367/s1, Supplementary File S1: Morphological matrix nexus file.
