# Correction: Szawaryn et al. A New Tribe of the Ladybird Beetle Subfamily Microweiseinae (Coleoptera: Coccinellidae) Discovered on an Island in the North Atlantic Ocean. *Insects* 2020, *11*, 367

**DOI:** 10.3390/insects15060460

**Published:** 2024-06-19

**Authors:** Karol Szawaryn, Jaroslav Větrovec, Wioletta Tomaszewska

**Affiliations:** 1 Museum and Institute of Zoology, Polish Academy of Sciences, Twarda 51/55, 00-818 Warsaw, Poland; 2 Buzulucká 1105, 50-003 Hradec Králové, Czech Republic; jerryvetrak@seznam.cz

## 1. Introduction

The recent paper by Szawaryn et al. (2020) published in *Insects*, 11 (367) [1], was not in full compliance with the *International Code of Zoological Nomenclature* [2] regarding the publication of online taxonomic papers. Article 8.5. states that to be considered published [within the meaning of the Code], “a work issued and distributed electronically must be registered in the Official Register of Zoological Nomenclature (ZooBank) (see Article 78.2.4) and contain evidence in the work itself that such registration has occurred” (Article 8.5.3). Because the paper by Szawaryn et al. (2020) was not registered in ZooBank prior to publication, and therefore evidence of registration was not included in it, the new taxonomic names proposed in the paper are not available under the Code [3]. This condition was also emphasized in the paper by Bouchard et al. [4]. The purpose of this paper is to make those names available.

To fulfill the requirements of Article 8.5 of the code, this paper has been registered in ZooBank, with the LSID above, and the names of the tribe, genus, and species described below have also been registered, following recommendation 10B of the Code. Their LSIDs are given under each name.

To meet the requirements of Article 13.1.2 of the Code, the names listed below are accompanied by a bibliographic reference to their full descriptions, and are thereby made available by the publication of this paper. As the wording of Article 13.1.2 is somewhat ambiguous regarding the status of descriptions based on bibliographic references, to avoid further problems, we have added below a brief description differentiating each taxon and a holotype designation with the repository identified; these are repeated from the original paper [1].

## 2. New Nomenclatural Acts

Order: Coleoptera Linnaeus, 1758.
Family: Coccinellidae Latreille, 1807.
Subfamily: Microweiseinae Leng, 1920.

**Tribe: Madeirodulini trib. nov.**

Madeirodulini Szawaryn, Větrovec and Tomaszewska 2020: 5 (not available)

Type genus. *Madeirodula* gen. nov., by monotypy and present designation.

ZooBank: urn:lsid:zoobank.org:act:B0157299-987D-4A22-8C62-E6D7B9AF6498

**Etymology.** The tribal name is derived from the name of a type genus.

**Diagnosis.** The new tribe Madeirodulini resembles members of the tribe Microweiseini in general body shape, but it can be separated from them by its bidentate mandibular apex (vs. unidentate), antennae consisting of 11 antennomeres (vs. 7–10), cultriform apical maxillary palpomere (vs. conical, parallel-sided, or rounded), paired apophyses of male sternum IX joined apically in form of inverted V (vs. inverted Y), and by a lack of line separating anterior corners of pronotum from disc. Bidentate mandibles, antennae with 11 antennomeres, and cultriform apical maxillary palpomere are shared with members of the tribe Carinodulini; however, Madeirodulini can be separated from Carinodulini by the prosternum that forms a large chinpiece, abdominal postcoxal lines descending and incomplete laterally (vs. U- or V-shaped in Carinodulini), well-developed hind wings, and by a lack of lateral carinae or pits on the pronotum. From Serangiini, Madeirodulini can be distinguished by having an antennal club consisting of three atennnomeres (vs. one antennomere), the mandible with a broadly rounded, simple external border (vs. projected into a process in Serangiini), and the elytral epipleura without foveae for the reception of legs. See Szawaryn, Větrovec and Tomaszewska, 2020: 5–7, Figures 2–4 [1] for a full description.

**Genus: *Madeirodula* gen. nov.**

*Madeirodula* Szawaryn, Větrovec and Tomaszewska 2020: 7 (not available)

Type species. *Madeirodula atlantica* sp. nov., by monotypy and present designation.

ZooBank: urn:lsid:zoobank.org:act: A09A18D3-BA7C-41BD-8F77-0D08A4027186

**Etymology.** The first part of the genus name is derived from the name of the island where the type specimens were collected, and the second part refers to *Carinodula*, the type genus of Carinodulini, which is a sister group of the new tribe.

**Diagnosis.** Same as for the tribe. See Szawaryn, Větrovec and Tomaszewska, 2020: 7–9, Figures 2–4 [1] for a full description.

***Madeirodula atlantica* sp. nov.**

*Madeirodula atlantica* Szawaryn, Větrovec and Tomaszewska 2020: 9 (not available)

ZooBank: urn:lsid:zoobank.org:act: 483232A5-650A-4567-B4EE-FD6B68DF6EB1

**Etymology.** The specific name refers to the Atlantic Ocean.

**Type material.** Holotype, male; Madeira, 16.11.2017, Santa Maria Madalena, Pombais, costal slopes, 32°51′31.8″ N 17°12′10.3″ W, 400 m, lgt. J. Větrovec. Paratype, male; same data as holotype. (Holotype repository: Natural History Museum Prague, Czechia, paratype: Jaroslav Větrovec collection.)

**Diagnosis.** Same as for the genus. See Szawaryn, Větrovec and Tomaszewska, 2020: 9–10, Figures 2–4 [1] for a full description.
